# The Late Evolution of the Nascent Peptide Code for Translational Control and Its Relationship to the Standard Genetic Code

**DOI:** 10.3390/genes17060619

**Published:** 2026-05-29

**Authors:** Gustavo Caetano-Anollés

**Affiliations:** 1 Department of Crop Sciences, University of Illinois, Urbana, IL 61801, USA; gca@illinois.edu; Tel.: +1-217-333-8172; 2 Carl R. Woese Institute for Genomic Biology, University of Illinois, Urbana, IL 61801, USA

**Keywords:** nascent peptide code, translational control, mRNA stability, dipeptides, genetic code evolution, phylogenomics, ribosome dynamics, protein evolution

## Abstract

**Background**: Recent work has revealed that protein-coding sequences encode regulatory information influencing mRNA stability and translation through a nascent peptide code. However, the evolutionary origin of this regulatory layer remains unclear. This study aims to determine when peptide-mediated translational control emerged during the evolution of the proteome and genetic code. **Methods**: Dipeptide-specific effects on mRNA stability and translation were integrated with a phylogenetic timeline of dipeptide emergence derived from dipeptide sequences across proteomes. Each of the 400 canonical dipeptides was assigned an evolutionary age, and experimentally derived regulatory effects were mapped onto this timeline, with associations assessed using rank-based correlation and regression analyses. **Results**: A weak but statistically significant negative association was observed between dipeptide age and mRNA stability, indicating that more recently evolved dipeptides tend to destabilize transcripts. This trend was stronger at the amino acid level, where later-emerging residues showed greater contributions to reduced mRNA levels. Destabilizing effects were associated with physicochemical properties such as positive charge, side-chain bulkiness, and β-strand propensity. Mapping these effects onto codon space revealed a non-random distribution aligned with the evolutionary and structural organization of the genetic code. Destabilizing effects were also enriched within specific codon exchange groups, indicating that regulatory signals are structured within the degeneracy and mutational neighborhoods of the code. **Conclusions**: These findings indicate that the nascent peptide code is a late evolutionary innovation linked to amino acid expansion and proteomic complexity, with regulation embedded within both peptide sequences and the degeneracy structure of the standard genetic code.

## 1. Introduction

A growing body of work has expanded the classical view of translational regulation—long centered on codon usage, tRNA availability, and untranslated region (UTR) elements—by implicating the protein-coding sequence itself as an active determinant of mRNA fate [1,2,3]. Studies in eukaryotes have shown that features embedded within coding regions, including codon optimality, GC content, and amino acid composition, can influence mRNA stability and translational efficiency [4,5,6,7,8]. However, disentangling the relative contributions of these factors has remained challenging, especially in human cells, where endogenous transcripts differ simultaneously in multiple sequence attributes and structural contexts [5,6,7,8,9,10,11].

A significant advance in resolving these issues is provided by Burke et al. [11], who systematically interrogated the effects of coding sequence motifs on mRNA stability using a massively parallel reporter assay in human cells. By assaying thousands of synthetic constructs representing all possible codon-pair combinations, the authors uncovered a previously unappreciated regulatory layer: a “nascent peptide code” embedded in the translated sequence itself. Their central finding is that specific dipeptide combinations—rather than individual amino acids or synonymous codons—can trigger marked reductions in mRNA stability. Sequences enriched in bulky and positively charged residues induce ribosome slowdown and translational arrest, consistent with earlier observations that polybasic nascent peptides interact with the ribosomal exit tunnel and impede elongation [12,13,14,15,16].

Mechanistically, these effects reinforce the emerging view that translational elongation dynamics are tightly coupled to mRNA stability through ribosome-associated quality control pathways [3,13,14,15,16,17,18]. Ribosome pausing induced by unfavorable sequence features can trigger surveillance mechanisms that lead to premature termination and accelerated transcript decay. However, prior work has typically attributed such effects to isolated variables—such as codon optimality or amino acid charge—without accounting for higher-order interactions among sequence features [4,5,6,7,8]. Burke et al. [11] resolve this limitation by demonstrating that the destabilizing effects of coding sequences are inherently combinatorial. They show that specific dipeptide motifs, rather than single residues, are predictive of mRNA instability, and that these motifs are strongly associated with structural properties of the nascent peptide. Destabilizing sequences exhibit a pronounced tendency to adopt extended β-strand conformations, linking secondary structure formation within the ribosomal exit tunnel to translational slowdown and mRNA decay. This finding establishes a direct connection between peptide biophysics and gene expression regulation that is not captured by models based solely on codon usage or amino acid composition.

From an evolutionary perspective, the nascent peptide code provides a mechanistic substrate for selection acting directly on short peptide motifs within coding regions. Because these motifs influence both translational dynamics and mRNA stability, they introduce feedback between protein sequence evolution and gene expression regulation. In this sense, the code represents an intrinsic layer of post-transcriptional control encoded within the proteome itself, complementing more traditional regulatory mechanisms.

In the present study, I build on this framework by mapping dipeptide-specific effects on mRNA stability and translation [11] onto a phylogenetic timeline of dipeptide evolution. This analysis leverages phylogenomic reconstructions of 400 canonical dipeptides across proteomes, which reveal a well-resolved tree of dipeptide sequences and a staged pattern of emergence linked to the expansion of amino acid usage and the transition from an early operational RNA code to the standard genetic code [19]. By integrating experimentally determined regulatory effects with inferred dipeptide ancestries, I reconstruct the temporal emergence of the nascent peptide code within this evolutionary context. This framework enables us to test whether peptide-mediated translational control is an ancient feature or a late innovation associated with increasing proteomic complexity and the incorporation of amino acids linked to anticodon-based coding [20]. The late emergence of specific dipeptide classes, together with the co-evolution of amino acid usage and properties such as protein thermostability [19], provides an evolutionary backdrop for situating the nascent peptide code. This phylogenetic integration addresses a central question left open by functional studies: when did peptide-mediated translational control arise, and how is its emergence related to the transition from an early operational code to the fully developed standard genetic code?

## 2. Materials and Methods

Dipeptide-specific effects on mRNA stability and translational efficiency were obtained from published massively parallel reporter assays that systematically evaluated all codon-pair combinations in human cells [11]. These datasets quantify the impact of individual dipeptides on mRNA abundance, ribosome translation rates, and translational output. Dipeptides were assigned quantitative scores reflecting their stabilizing or destabilizing effects on mRNA levels. mRNA levels were expressed as log_2_ fold-change (LFC) values derived from reporter assays, where values represent relative changes in steady-state mRNA abundance compared to a reference. Negative values imply destabilizing/reduced mRNA levels, while positive values imply stabilizing/increased mRNA levels. When multiple synonymous codons encoded the same dipeptide, values were averaged to obtain amino acid–level effects, thereby removing codon bias and isolating peptide-level contributions. Dipeptide effects were quantified using average log_2_ fold-change (avg_LFC) in mRNA abundance as provided in the source dataset. All mRNA stability measurements were from reporter assays performed in human cells [11].

Evolutionary ages of dipeptides were derived from a phylogenomic tree of dipeptide sequences (ToDS) reconstructed from normalized abundance profiles of 400 canonical dipeptides across 1561 proteomes spanning the three superkingdoms of life [19]. Phylogenetic inference was performed using maximum parsimony, and evolutionary distance was expressed as node distance (*nd*), ranging from 0 (most ancient) to 1 (most recent). Each dipeptide was assigned an *nd* value representing its inferred time of origin as previously defined [19].

Dipeptide-specific regulatory scores were mapped onto their corresponding evolutionary ages by matching each dipeptide to its phylogenetic counterpart using standard one-letter amino acid codes to ensure unambiguous correspondence between datasets. The resulting dataset consisted of paired values (*nd_i_*, *R_i_*), where *nd_i_* is the evolutionary age of dipeptide *i*, and *R_i_* the measured regulatory effect. This integration enabled construction of a two-dimensional representation of regulatory impact across evolutionary time.

Associations between dipeptide age and regulatory effects were evaluated using Spearman’s rank correlation (ρ) and Pearson correlation; normality of variables was assessed using the Jarque–Bera and Anderson–Darling tests. Trends were further examined using linear and non-linear regression models. Differences between early (low *nd*) and late (high *nd*) dipeptides were assessed using the Mann–Whitney U test, given the non-normality of effect size distributions.

Finally, dipeptides were annotated with physicochemical and structural properties, including amino acid charge, side-chain bulkiness, hydrophobicity, and secondary structure propensity (α-helix and β-strand), based on standard scales. Associations among these features, regulatory effects, and evolutionary age were evaluated using correlation analyses and regression models. Data were visualized using scatter plots, kernel density plots, and heat maps, with sliding window averaging applied to dipeptides ordered by *nd* to compute local mean mRNA effects across neighboring observations. All analyses were conducted in R (v4.3) using custom scripts.

## 3. Results

### 3.1. Late Evolutionary Emergence of Dipeptide Regulatory Effects

**Figure 1:**
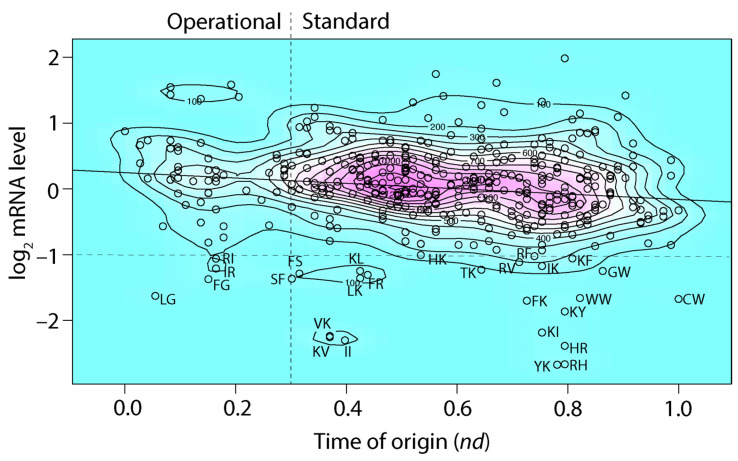
Bivariate kernel density plot describing a monotonic relationship between steady state log_2_ mRNA levels (LFC, arbitrary units) measured in human-cell reporter assays and time of origin (*nd*) of 400 dipeptides showing a weak negative Spearman’s rank correlation (ρ = −0.172; 2-sided *p*-value = 5.6 × 10^−4^; *S* = 12,498,873; *n* = 400). Kernel bandwidths were 0.055 (*nd*) and 0.160 (mRNA level). Scatterplots of ranks describe how rank transformations diminish homoscedasticity effects. The rise of the standard genetic code, ~3.0 billion years ago, is marked by a dashed vertical line. Dipeptides responsible for mRNA levels ≤ −1 are labeled.

To determine whether peptide-mediated translational control is an early or late evolutionary innovation, we mapped dipeptide-specific effects on steady-state mRNA levels onto a phylogenetic timeline of dipeptide emergence. This analysis revealed a statistically significant but weak negative monotonic relationship between dipeptide age and mRNA stability (Spearman’s ρ = −0.172), indicating that more recently evolved dipeptides tend to be associated with lower mRNA levels (Figure 1, Table S1). Because both variables significantly deviated from normality (Jarque–Bera and Anderson–Darling tests, *p* < 0.001), rank-based correlation was used as the primary measure of association.

Although the magnitude of the correlation is modest, the directionality of the relationship is consistent across the dataset and robust to non-parametric testing. Kernel density visualization highlights an enrichment of destabilizing dipeptides at higher node distance (*nd*) values, corresponding to later evolutionary stages. Early emerging dipeptides (low *nd*) cluster preferentially within regions of higher mRNA stability, whereas destabilizing effects are disproportionately represented among younger dipeptides. Highly destabilizing dipeptides, which include for example Lys, Val or Ileu, Ser and Phe, or Leu and Lys, emerged after *nd* = 0.3 (~3 billion years ago), which marks the onset of both the standard genetic code [19] and the formation of a fully functional ribosomal core [21]. These dipeptides form extended β strands that slowed ribosomal elongation and decreased mRNA levels over 8-fold relative to α-helix-promoting dipeptides [11].

### 3.2. Amino Acid Emergence Predicts mRNA Destabilization

**Figure 2:**
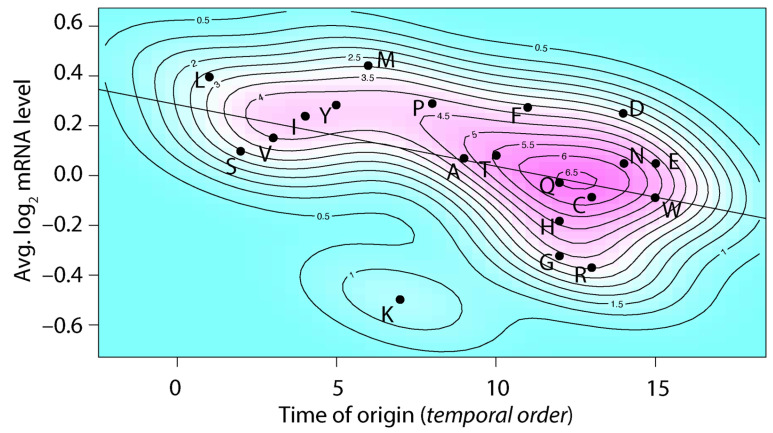
Bivariate kernel density plot describing a monotonic relationship between average steady state log_2_ mRNA levels of amino acids (avg_LFC, arbitrary units) measured in human-cell reporter assays and their time of origin (scale describing temporal order), showing a moderate negative Spearman’s rank correlation (ρ = −0.536; 2-sided *p*-value = 0.0148; *S* = 12,042.9; *n* = 20). Kernel bandwidths were 2.17 (time of origin*)* and 0.144 (mRNA level). Scatterplots of ranks describe how rank transformations diminish homoscedasticity effects.

To determine whether the observed dipeptide trends reflect deeper biochemical constraints, we examined the relationship between amino acid properties and their inferred times of origin. In contrast to the weak signal observed at the dipeptide level, amino acid-level analysis revealed a moderate negative monotonic association between average mRNA effects and evolutionary age (Spearman’s ρ = −0.536) (Figure 2). Because amino acid data satisfied normality assumptions (Jarque–Bera and Anderson–Darling tests, *p* > 0.14), Pearson correlation analysis further supported this trend (r = −0.44; *n* = 20), indicating that approximately 19% of the variance in mRNA stability can be explained by evolutionary ordering. Although this relationship approached but did not reach conventional statistical significance (two-sided *p* = 0.054; 95% CI: −0.74 to 0.01), the consistency between rank-based and parametric analyses supports a biologically meaningful signal.

Importantly, this stronger association at the amino acid level suggests that the late emergence of destabilizing dipeptides is rooted in the delayed incorporation of specific amino acid chemistries into the genetic code. Amino acids that contribute to translational slowdown—such as those with bulky side chains or positive charge—tend to appear later in evolutionary reconstructions, providing a mechanistic basis for the temporal pattern observed at the dipeptide level. That is the case of positively charged Lys, Arg and His.

### 3.3. Codon Organization and Exchange Groups Structure Regulatory Effects

**Figure 3:**
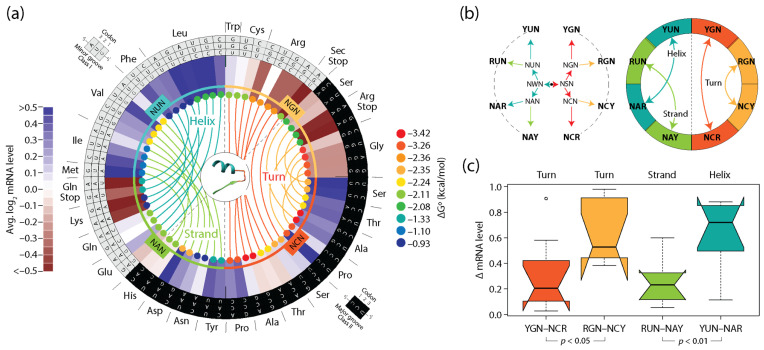
The nascent peptide and standard genetic codes. (**a**) Tracing average steady state log_2_ mRNA levels onto a circular genetic code compatible with energetics, complementarity and evolutionary history. Codons in grey boxes involve minor groove recognition (Class I) and those in black boxes involve major groove recognition (Class II). The four major quadrants divide the code according to the second codon position: NGN and NCN (orange and red) code for semipolar amino acids and are enriched in strong codon–anticodon base pairing interactions for positions 1 and 2 dimers (Turner energies of ΔG ≤ −2.36 kcal/mol), and NAN and NUN (green and aqua blue) code for hydrophilic and hydrophobic amino acids, respectively, and are enriched in weak interactions (ΔG ≥ −1.33 kcal/mol). Sectors with codons that are reverse complementary to each other are made explicit with lines of the same color connecting sense–antisense codon pairs. The networks define three sense–antisense codon exchange groups that retain secondary structure information in proteins [22]. Amino acids associated with the NSN half of the circular code are preferentially linked to turn motifs in protein structures, while the amino acids of the NWN half are linked to helix and strand structures. (**b**) The 8 sectors of the circular code are defined by a decision tree induced by ambiguity reduction and tRNA identity elements responsible for amino acid charging and coding specificities that matches tRNA and domain history [19,20]. Sense–antisense codon exchange groups are established by pairwise interactions between these sectors. (**c**) Notched box plots describing differentials of mRNA levels (ΔmRNA level) for sense–antisense codons of the four sense–antisense codon exchange groups (YGN–NCR, RGN–NCY, RUN–NAY, YUN–NAR) reveal a yin–yang pattern.

To place findings within genetic code structure, we mapped amino acid-level mRNA effects onto a circular representation of codon space that integrates codon–anticodon energetics, complementarity, and evolutionary history (Figure 3). This mapping reveals that amino acids associated with reduced mRNA stability are not randomly distributed but are preferentially localized within specific regions of the code, especially the NAR, YGN and RGN sectors. Late emerging amino acids with stronger destabilizing effects and bulky constitutions (e.g., Lys, Phe and His) are enriched in NAR and NAY sectors associated with weaker codon–anticodon interactions and with structural propensities for β-strand and extended conformations. These regions correspond to codon groups in which translational dynamics are more susceptible to perturbation, consistent with models of ribosome pausing and collision-mediated quality control. Conversely, late emerging destabilizing amino acids that are positively charged or have simpler physicochemical constitutions (e.g., Arg, Gly) are predominantly located in regions of the code characterized by stronger base-pairing interactions and sectors (YGN and RGN) linked to structural motifs such as turns.

Remarkably, the relationship between the sense and antisense use of codons, originally identified by Zull and Smith [22] as codon exchange groups preferentially associated with turn, helix and strand regions of protein structures, appears to be linked to mRNA destabilizing effects (Figure 3). Notched box plots describing differentials (Δ) of mRNA levels for sense–antisense codons of the four sense–antisense codon exchange sectors of the circular code (YGN–NCR, RGN–NCY, RUN–NAY, YUN–NAR) showed significant differentials with medians and averages ranging from 0.204 to 0.721 and from 0.250 to 0.642, respectively. They also showed a yin–yang pattern, with values for helix (YUN–NAR involving Lys, Leu and Phe) significantly larger (Wilcoxon rank test, two-tailed, *p* < 0.01) than those for strand (RUN–NAY involving His, Val and Ile), and those for turn comparable but larger (Wilcoxon rank test, two-tailed, *p* < 0.05) for RGN–NCY (involving Gly and Ser) than those for YGN–NCR (involving Arg, Cys, Ser and Thr). Note that notches indicating 95% confidence intervals for the medians do not overlap, suggesting statistically significant differences between their medians.

## 4. Discussion

### 4.1. Late Emergence of Peptide-Mediated Translational Control

The analyses presented here reveal a consistent evolutionary signal linking the emergence of dipeptides and amino acids to their effects on mRNA stability. At both levels of organization, more recently evolved sequence elements are associated with reduced steady-state mRNA levels, a pattern that is statistically supported and biologically coherent despite modest effect sizes at the dipeptide level (Figure 1 and Figure 2). This finding has direct implications for the origin of the nascent peptide code. Rather than representing a primordial feature of translation, the ability of short peptide motifs to modulate ribosome dynamics and mRNA decay appears to be a derived property that emerged relatively late in proteome evolution with the rise of the ribosome. Early emerging dipeptides are largely associated with neutral or stabilizing effects, whereas destabilizing motifs—those capable of triggering ribosome slowdown and transcript decay—are enriched among younger sequence elements. This temporal asymmetry supports a model in which translational control via nascent peptide interactions was not intrinsic to the earliest coding systems but instead arose as proteomes diversified and acquired greater physicochemical complexity [11].

### 4.2. Physicochemical Basis of Destabilization

The stronger evolutionary signal observed at the amino acid level suggests that the late appearance of regulatory dipeptides is rooted in the progressive incorporation of specific amino acid chemistries into the genetic code. Amino acids associated with destabilizing effects tend to possess properties known to interfere with ribosome translation rates, including positive charge, side-chain bulkiness, and propensity to form extended β-strand conformations. Experimental studies have shown that polybasic and bulky residues can interact unfavorably with the ribosomal exit tunnel, promoting translational pausing, ribosome collisions, and activation of quality control pathways such as ribosome-associated quality control (RQC) and no-go decay (NGD) [12,13,14,15,16,17,18]. These mechanisms couple elongation kinetics to mRNA stability, providing a direct route by which peptide sequence can influence transcript fate [1,2,3,8,9]. The present analysis focuses on dipeptides because they constitute the smallest experimentally tractable units that can be systematically analyzed across the complete canonical sequence space [11]. More complex arrest peptides, such as SecM and ErmC, likely represent specialized and context-dependent elaborations of the same general principle of nascent peptide-mediated translational regulation [23,24,25]. In this framework, dipeptide effects may be viewed as elementary components upon which larger regulatory motifs evolved. Polyproline motifs were not strongly enriched among the destabilizing dipeptides identified here. This likely reflects mechanistic differences between polyproline-dependent translational pausing and the peptide-mediated effects associated with bulky and positively charged residues. Whereas polyproline stalling primarily arises from inefficient peptide bond formation and dependence on specialized elongation factors such as EF-P or eIF5A [26,27,28], the destabilizing motifs of this study are more closely associated with electrostatic and structural interactions within the ribosomal exit tunnel.

Within this framework, the nascent peptide code emerges as a higher-order property of sequence composition: it reflects not only the presence of specific residues but also their combinatorial arrangement into dipeptides that encode structural and electrostatic features capable of modulating ribosome behavior [11]. The enrichment of such motifs among late-emerging dipeptides indicates that the regulatory capacity of the code depends on evolutionary innovations in amino acid usage.

### 4.3. Integration with Genetic Code Evolution

The evolutionary timing inferred here aligns with models proposing a stepwise expansion of the genetic code, in which early coding systems relied on a restricted set of amino acids with relatively simple physicochemical properties [19,20]. These early amino acids are thought to have been specified through operational interactions in tRNA acceptor stems, preceding the full establishment of anticodon-mediated coding [20].

In this context, the absence of strong destabilizing effects among early dipeptides is expected. A limited amino acid repertoire would constrain the range of possible peptide structures and reduce the likelihood of sequences capable of inducing ribosomal stress. As the code expanded to include additional amino acids—particularly those with charged or bulky side chains—the combinatorial space of dipeptides increased dramatically, enabling the dissection of motifs with regulatory potential.

The mapping of mRNA stability effects onto the circular genetic code (Figure 3) further supports this interpretation. Amino acids associated with destabilizing effects are preferentially located in regions of codon space characterized by weaker codon–anticodon interactions and distinct structural propensities. This spatial organization suggests that the evolution of the genetic code not only expanded the chemical repertoire of proteins but also introduced new layers of regulatory complexity embedded within coding sequences. Importantly, mapping mRNA stability effects onto codon exchange groups (Figure 3) shows that regulatory signals extend beyond amino acid identity and are structured within the degeneracy of the genetic code. Dipeptides associated with reduced mRNA stability are enriched in specific codon exchange groups, indicating that synonymous and near-synonymous relationships constrain the distribution of regulatory effects. This pattern suggests that the nascent peptide code is embedded within mutational neighborhoods defined by the genetic code, linking peptide-mediated translational control to codon-level evolutionary dynamics. This codon-centric organization further indicates that destabilizing peptide motifs did not arise independently but co-evolved with codon interchangeability and redundancy. Consequently, regulatory information encoded in nascent peptides reflects both amino acid physicochemical properties and the topology of codon space, providing a mechanistic bridge between nucleotide- and peptide-level evolution.

The association between destabilizing regulatory effects and amino acid properties such as positive charge, side-chain bulkiness, and β-strand propensity also connects the present findings to coevolutionary models of genetic code origin. Previous studies have proposed that the organization of the genetic code reflects not only biosynthetic relationships among amino acids, but also the optimization of physicochemical and structural properties relevant to protein folding and stability [29,30,31]. β-structural propensities and partition–energy relationships have been suggested to influence the evolutionary distribution of amino acids within codon space [29,30].

Within this framework, the late emergence of destabilizing peptide motifs may reflect the progressive incorporation of amino acids associated with increasingly differentiated structural and energetic functions in proteins. The observed enrichment of regulatory effects within specific regions of codon space and codon exchange groups is therefore consistent with the idea that the evolution of the genetic code integrated biosynthetic accessibility, protein structural constraints, and translational regulatory potential into a unified coding architecture.

### 4.4. Proteome Complexity and Translational Regulation

The late emergence of the nascent peptide code can also be viewed in the broader context of increasing proteome complexity. As proteins evolved longer sequences, more diverse folds, and more intricate functional domains, the demands on translational fidelity and quality control would have intensified. Mechanisms linking elongation dynamics to mRNA stability provide an efficient means of monitoring translation in real time, allowing the cell to detect and resolve problematic sequences [3,12,13,14,15,16,17,18]. The late emergence of destabilizing peptide motifs may also have coincided with the evolution of translational rescue and elongation-support systems, including EF-P/eIF5A and ABCF-family proteins involved in ribosome protection and stall resolution [32,33]. Such co-evolution would be consistent with increasing proteomic complexity and the growing need to manage ribosome pausing associated with structurally challenging peptide sequences.

In this scenario, the nascent peptide code represents an adaptive integration of structural and regulatory information within protein-coding sequences. Rather than being imposed externally, regulatory signals are encoded directly in the physicochemical properties of the nascent peptide. This integration enables fine-tuned control of gene expression while simultaneously shaping the evolution of protein sequences through selective pressures acting on both function and translational efficiency [4,5,6,7].

The association between regulatory effects and codon exchange groups further suggests that translational control may have been shaped by constraints on mutational accessibility. Because codon exchange groups define pathways of permissible substitutions [34,35], the enrichment of destabilizing motifs within particular groups indicates that evolutionary trajectories through sequence space may bias the emergence of regulatory features [36]. This coupling between mutational structure and regulatory outcome provides an additional layer of integration between the genetic code and the nascent peptide code.

### 4.5. Limitations and Interpretative Scope

While the evolutionary trends identified here are statistically robust, the relatively weak correlation observed at the dipeptide level indicates that the nascent peptide code is only one of several factors influencing mRNA stability. Codon usage, RNA structure, translation initiation rates, and cellular context all contribute to the overall regulation of gene expression [2,4,5,6,7,8,9,10]. Moreover, the experimental measurements used to quantify dipeptide effects are derived from controlled reporter systems, which may not fully capture the complexity of endogenous transcripts. The integration of these data with phylogenetic reconstructions introduces additional sources of variability, including uncertainty in the inferred timing of dipeptide emergence [19]. Despite these limitations, the consistency of the observed trends across levels of analysis, together with their mechanistic plausibility, supports the conclusion that evolutionary history has shaped the distribution of peptide-mediated regulatory signals in modern proteomes.

## 5. Conclusions

The late emergence of peptide-mediated translational control has important implications for understanding the origin of the standard genetic code. If early coding systems lacked the capacity for peptide-driven regulation of mRNA stability, then the primary selective pressures shaping the early code were likely related to basic biochemical functionality and translational efficiency, rather than regulatory complexity [19,20]. The subsequent incorporation of amino acids capable of generating destabilizing peptide motifs would have introduced new constraints on coding sequences, linking protein composition to gene expression outcomes. This transition marks a shift from a primarily structural and catalytic role of proteins to a more integrated role in cellular regulation.

In this light, the nascent peptide code can be viewed as a secondary layer of information superimposed on the genetic code, emerging only after the core mapping between codons and amino acids was established. Future work integrating codon-level, structural, and phylogenetic data will further clarify how regulatory information became embedded within the genetic code.

The integration of mRNA stability effects with codon exchange group structure further indicates that the nascent peptide code is shaped not only by amino acid chemistry, but also by constraints imposed by codon organization and exchangeability. Together, these results support a model in which the nascent peptide code emerged late but became tightly integrated with the genetic code, linking protein sequence evolution, codon structure, and gene expression regulation into a unified evolutionary framework.

## Data Availability

The original contributions presented in this study are included in the article/Supplementary Materials. Further inquiries can be directed to the corresponding author.

## Supplementary Materials

The following supporting information can be downloaded at https://www.mdpi.com/article/10.3390/genes17060619/s1, Table S1: Data analysis in xlsx format.
